# Effectiveness of tumescent solution combined with negative pressure wound therapy in traditional high ligation and stripping of the great saphenous vein

**DOI:** 10.1097/MD.0000000000019040

**Published:** 2020-03-13

**Authors:** Feng Su, Liu Cheng, Qiao Tong

**Affiliations:** a Department of Vascular Surgery, The Xuzhou School of Clinical Medicine of Nanjing Medical University; b Department of Vascular Surgery, Xu Zhou Central Hospital, Xuzhou; c Department of Vascular Surgery, Drum Tower Clinical Medicine College of Nanjing Medical University; d Department of Vascular Surgery, The Affiliated Drum Tower Hospital of Nanjing University Medical School, Nanjing, China.

**Keywords:** ecchymosis, high ligation and stripping of great saphenous vein, pain, tumescence solution

## Abstract

Traditional high ligation and stripping (THLS) is a routine operation for varicose veins. However, THLS is accompanied with postoperative subcutaneous ecchymosis and pain. In this current study, we aimed to explore the effect of tumescence solution (TS) combined with negative pressure wound therapy (NPWT) on the relief of subcutaneous ecchymosis and pain after THLS of great saphenous vein.

A total of 180 patients receiving THLS were enrolled in group A, and 120 patients undergoing THLS and TS combined with NPWT were assigned into group B. The occurrences of subcutaneous ecchymosis and pain were recorded. Moreover, the total area of subcutaneous ecchymosis was estimated by the grid method. Visual analogue scale (VAS) score was used to assess the pain level of both groups.

Preoperative characteristics were not significantly different between 2 groups. Postoperative ecchymosis occurred in 112 cases (62.2%) of group A and 41 cases (34.2%) of group B. The area of ecchymosis in group A (66.6 ± 44.5) cm^2^ was larger than that in group B (25.2 ± 19.9) cm^2^. The number of patients without obvious pain in group A (57, 31.7%) was significantly less than that in group B (77, 64.2%) after operation. In addition, VAS score in group A (3.1 ± 2.6) was higher than that in group B (2.2 ± 1.9).

In conclusion, the application of TS combined with NPWT in THLS can not only alleviate subcutaneous ecchymosis and pain, but also prevent the occurrence of subcutaneous ecchymosis and pain after operation. Therefore, it is conducive to postoperative recovery and is suitable for clinical application.

## 1 Introduction

At present, traditional high ligation and stripping (THLS) is the most common surgical intervention to treat varicose veins globally.^[1]^ Despite the recent emergence of laser, radio-frequency, foam sclerotherapy, and other novel therapeutic modalities, THLS is still the dominant option to treat varicose veins of the lower extremities.^[2–4]^ However, subcutaneous ecchymosis and pain discomfort may occur in the thighs of some patients after THLS. Although subcutaneous ecchymosis can be absorbed and recovered by itself and pain can be treated with drugs, these 2 postoperative complications still exert certain side effect on patients after operation.^[5,6]^ Tumescence solution (TS) is a mixture of highly diluted lidocaine and adrenaline, which can infiltrate the tissue around the veins to further exert a certain analgesic and oppressive effect.^[7]^ After the thigh trunk of great saphenous vein (GSV) is stripped, the vascular tunnel wound is left, followed by insertion of the drainage tube with side hole into suction with negative pressure, thereby playing a sustained negative pressure wound therapy (NPWT) and compression role. In order to further explore possible approaches of attenuating subcutaneous ecchymosis and pain after THLS, the infiltration of TS around the GSV in thigh was combined with NPWT on the wound of the vascular tunnel after stripping. We further investigated whether the combined therapy played an active role in promoting the postoperative recovery of patients.

## 2 Materials and methods

### 2.1 Patients

A total of 300 patients with varicose veins of the lower extremity were retrospectively collected, who were admitted to the Vascular Surgery Department of Xu Zhou Central Hospital from January to December 2015. The diagnostic criteria were as follows: all patients were treated with a single limb for 1 operation, according to the International Venous Alliance CEAP clinical grade is C3 to C6; deep vein thrombosis and stenosis or compression of the iliac vein were ruled out by deep vein venography, atrial septal defect and pulmonary hypertension were excluded by cardiac echocardiography; patients with coagulopathy, lower extremity infection, oral anticoagulation or antiplatelet drugs, or older than 65 years were eliminated. The included cases were divided into 2 groups: routine THLS patients as the control group (N = 180 cases for group A), intraoperative use of TS combined with NPWT treatment for the observation group (N = 120 cases for group B).

This study conformed to the ethics standard from the Ethics Committee of Xu Zhou Central Hospital.

### 2.2 Surgical methods

**Figure 1:**
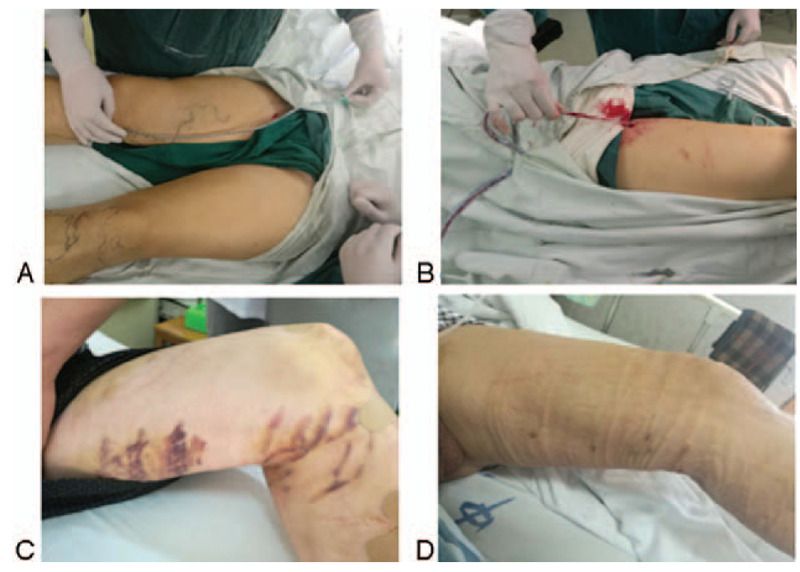
A. Negative pressure drainage silicone tube with side hole was placed on the surface of the vascular bed tunnel. B. Silicone tube was placed in the vascular bed tunnel of the thigh after THLS of the great saphenous vein. C. Subcutaneous ecchymosis was obvious after THLS, mostly concentrated in the thigh (Group A). D. No obvious subcutaneous ecchymosis was observed after THLS in patients treated with TS infiltration combined with NPWT (Group B). NPWT = negative pressure wound therapy, THLS = traditional high ligation and stripping, TS = tumescence solution.

All patients were treated with intravenous stripping guide wire (Beijing Puyi Shengji Technology Co., Ltd., Beijing, China). Patients in the control group received traditional THLS, while patients in the observation group were treated with TS (containing 1 mg adrenaline and 100 mg ropivacaine in 500 mL saline) following the performance of traditional stripping of GSV. The exact dosage of TS used for each leg depending on the length of the thigh. When the venous exfoliation guide wire entered GSV trunk, the subcutaneous tissue was infiltrated with a fine needle by TS at a distance of 1 cm from the 2 sides of the GSV. The thigh trunk of GSV was subsequently stripped by vein exfoliation guide wire. Silica tube with side hole was pulled and placed in the wound of the thigh vascular tunnel of GSV. NPWT was applied at a pressure of 200 mmHg for 20 minutes. Patients of both groups got out of bed on the first day after surgery. The elastic bandage was wrapped for 48 hours, followed by the administration of elastic stockings (Fig. 1A and B).

### 2.3 Ecchymosis

The skin ecchymosis was observed and measured on the third postoperative day after the removal of the skin bandage. In this study, only skin ecchymosis of the thigh above the knee joint was observed. Skin ecchymoses with an area >1 cm^2^ and more obvious color were observed and recorded, with the total area (cm^2^) estimated by the square method. Skin ecchymosis not noticeable in color or scattered distribution or area <1 cm^2^ were not included. In the case of multiple ecchymosis, the areas of them were added. Observations, measurements, and records of all skin ecchymosis were performed by the same experienced nurse. The postoperative ecchymosis in patients of the 2 groups was shown in (Fig. 1C and D).

### 2.4 Pain

All patients were recorded for pain score. Visual analogue scale (VAS) method was used to measure the pain level on the first day after surgery. In visual analogue scoring, 10 cm long vernier with 10 scales on one side were used, with “0” and “10” on both ends, 0 for pain, and 10 for the most intense pain that was unbearable. Patients were asked to select the appropriate location based on their subjective feelings, and the nurse read the score according to the scale. If the patient required analgesic medication due to severe pain, VAS should be scored before the administration of medication. Similarly, pain VAS score of the patient was collected by the same nurse on the first day after surgery.

### 2.5 Statistical methods

SPSS 18.0 software (SPSS Inc., Chicago, IL, USA) was used for statistical analysis. Measurement data following or approximating normal distribution were shown as mean ± standard deviation (mean ± SD). Independent sample *t* test was used for comparison between groups; Chi-squared test was used for counting data between the 2 groups. A *P* value <.05 was considered as statistical significance.

## 3 Result

### 3.1 General preoperative data

A total of 300 cases were retrospectively enrolled in this study, all of whom had smooth surgical procedures. There were 87 female patients and 93 men in group A, 49 women and 71 men in group B; the mean age of group A and group B was 40.9 ± 6.2 years old and 39.7 ± 7.3 years old, respectively; the average body mass index of group A and group B was 24.9 ± 3.9 and 25.5 ± 4.1, respectively; the saphenous vein trunk at saphenous femoral vein junction in group A and group B was 9.7 ± 4.1 and 9.3 ± 3.8 mm, respectively. There was no significant difference in age, sex, body mass index, saphenous vein trunk at saphenous femoral junction and clinical classification between the 2 groups before operation. The detailed preoperative data of the 2 groups were shown in Table 1.

**Table 1:**
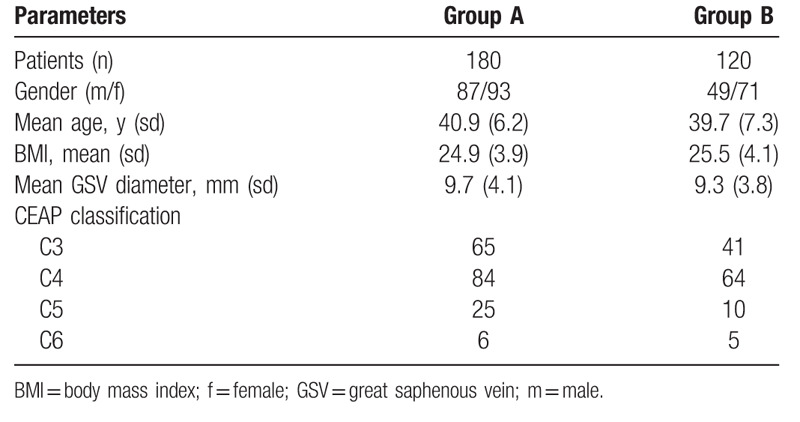
Preoperative characteristics of 2 groups of patients.

### 3.2 Ecchymosis evaluation

The occurrence and area of thigh ecchymosis were recorded in the third day after surgery. There were 112 cases of ecchymosis in group A (62.2%) and 41 ones in group B (34.2%). The area of ecchymosis was 66.6 ± 44.5 cm^2^ in group A, and was 25.2 ± 19.9 cm^2^ in group B. The proportion of ecchymosis cases was obviously decreased and the area of ecchymosis was considerably attenuated after operation in group B than those in group A (Tables 2 and 3).

**Table 2:**
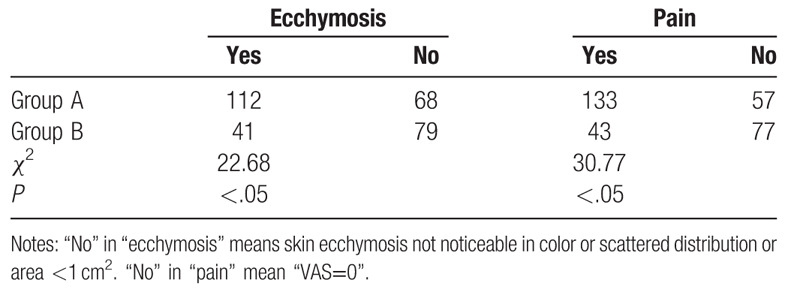
Postoperative ecchymosis and pain in 2 groups of patients.

**Table 3:**
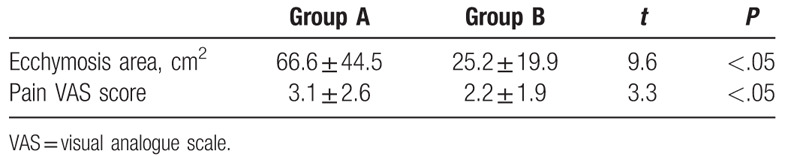
Postoperative subcutaneous ecchymosis area and pain VAS score.

### 3.3 Pain evaluation

VAS score was recorded on all patients on the first day after operation. As a result, there were 57 patients (31.7%) without pain in group A, 77 patients (64.2%) in group B. The VAS score of group A and group B was 3.1 ± 2.6 and 2.2 ± 1.9, respectively. The VAS score was significantly improved in group B compared to that in group A (Tables 2 and 3).

## 4 Discussion

Varicose veins of lower extremities are common diseases. In spite of multiple new therapies for varicose veins of lower extremities, THLS is still the mainstream surgical method for varicose veins from C3 to C6 due to its exact curative effect and low cost.^[8,9]^ In addition, diverse novel technologies are applied on the basis of classical surgery.^[10]^ However, THLS can cause excessive tissue trauma due to stripping of blood vessels, leading to complications such as the femoral vein injury, postoperative pain and numbness, subcutaneous ecchymosis and hematoma.^[11–13]^ According to previous literature, postoperative subcutaneous ecchymosis can reach to approximately 80%.^[14]^ Postoperative complications, such as pain and ecchymosis, are not serious disorders, which can be alleviated or cured by appropriate treatment. However, these complications could prolong the hospitalization time of patients, increase medical burden, greatly affect patients’ medical experience, exert a certain impact on patients’ psychology, and even become the disputed point of doctor–patient contradiction. At present, most hospitals or departments focus more on learning and developing new technologies, while neglect traditional operations, paying less attention to the details of reducing postoperative complications. In this study, TS combined with NPWT was applied to THLS to observe its effect on attenuating pain and skin ecchymosis after operation, and to further reduce the complications of THLS. As a result, we demonstrate that this combined therapy has significant effect in attenuating the subcutaneous ecchymosis and pain after THLS, with definite clinical value.

TS is commonly used in plastic surgery, vascular surgery, breast surgery, etc,^[15–18]^ which has the advantage of reducing bleeding and pain. TS is defined as a mixed solution of adrenaline and local anesthetic drugs in a certain proportion and concentration. The contraction effect of adrenaline on small blood vessels and the swelling effect on the vein serve as a good hemostasis.^[19]^ A combination of short-acting lidocaine and long-acting ropivacaine can decrease pain, which work well together and are regarded as the main cause of postoperative pain relief. Swelling infiltration of thighs and NPWT of blood vessel tunnels are of great use in pain relief and hemostasis. First, the perivenous tissue is anesthetized, and the anesthesia fluid infiltrates along the space around the GSV to exert certain anesthetic effects on the peripheral nerves and to effectively reduce postoperative pain. Second, the infiltration of TS along the main saphenous vein can play a role in compressing the tissue blood vessels. And NPWT can compress the tissue inside the blood vessel tunnel, and further compression can reduce bleeding. In addition, complete hemostasis measures can also reduce the pain stimulus caused by bleeding.

In 1987, Klein first infiltrated a large number of diluted adrenaline-containing lidocaine solution into the subcutaneous fat layer for local fat aspiration anesthesia, namely swelling anesthesia.^[20,21]^ Due to its safety, less blood loss, less tissue damage, prolonged anesthesia time, and good analgesic effect, it has been widely used in various plastic operations ever since.^[19]^ Comparatively, patients in maxillofacial surgery and plastic surgery often have higher requirements for reducing pain and maintaining beauty. TS is therefore widely utilized, showing the excellent advantages and worthwhile reference by vascular surgeons. At present, TS is widely used to treat varicose veins of the lower extremities by radiofrequency and laser.^[22]^

Although TS is used reduce thermal damage in endovenous radiofrequency and intravenous laser treatment of varicose veins of lower extremities,^[23,24]^ there are still concomitant thigh pain and subcutaneous ecchymosis after surgery.^[25–27]^ Ecchymosis is defined as a large amount of discoloration caused by blood infiltration into the subcutaneous tissue.^[28]^ During high ligation and stripping of the GSV, the collateral veins are torn apart after stripping the GSV trunk, thus, part of the veins are retracted into the tissue due to shrinkage of the blood vessels, which is considered as the main cause of subcutaneous ecchymoses. Postoperative pain is directly related to vascular tunneling in the saphenous vein. Infiltration of TS is of great value in tissue swelling, which can effectively reduce postoperative bleeding and pain. It is generally conceived that the infiltration of TS around the saphenous vein trunk has begun before the beginning of saphenous vein trunk treatment, and soon begins to spread, which may reduce the role of swelling effect. In order to attenuate the disadvantage of rapid disappear of swelling action, silica gel tube with side holes is inserted into the vascular tunnel after stripping of saphenous vein trunk of thigh in the process of traditional stripping of saphenous vein trunk, which can play a good compressive role to enhance the swelling function.

Wound vacuum suction is applied in various departments,^[29]^ especially in trauma surgery, which can achieve a good wound hemostasis.^[30]^ The application of negative pressure on the wound surface can not only stop the bleeding but also effectively remove errhysis and seepage. Therefore, it is very common in orthopedics and trauma surgery,^[31]^ with certain advantages in treating wounds.^[32]^ NPWT can effectively remove the exudate from the wound, thereby reducing the influence of exudate on wound recovery after operation. The vacuum suction of the wound is often flushed by the suction and drainage fluid. In this study, we mainly focus on tissue compression caused by vacuum suction. NPWT is often used to wash the wound by suction plus drainage fluid, and we focus on tissue compression produced by NPWT. The application of negative pressure drainage tube in GSV stripping can obviously play a role in vascular tunnel compression and hemostasis to timely attract blood seepage in the tunnel, which is of great significance in postoperative recovery.

TS cannot fully compress the vascular tunnel left by the saphenous vein exfoliation, which can be compensated by vacuum suction. Indeed, the infiltration of TS into the surrounding tissue of the wound and NPWT of the wound can effectively perform the full compression and hemostasis on the surgical scope tissue. The special local anesthesia effect of TS and the advantage of NPWT in removing the exudate from wound surface have good analgesic effect and can promote recovery after operation. Thus, the combination of the 2 methods is considered as the main cause of postoperative relief in ecchymosis and pain.

In summary, postoperative subcutaneous ecchymosis and pain may occur in many patients after THLS. Currently, only drugs and physiotherapy are available for relief. In this study, we show that the occurrence of subcutaneous ecchymosis and pain can be reduced or even prevented by intraoperative intervention. In THLS, a combination of TS with NPWT can effectively reduce the pain and subcutaneous ecchymosis, which is safe, reliable and satisfactory. Hopefully, it can play a positive role in the postoperative recovery of patients.

## Author contributions

**Conceptualization:** Qiao Tong.

**Data curation:** Feng Su, Liu Cheng.

**Formal analysis:** Feng Su, Liu Cheng.

**Funding acquisition:** Qiao Tong.

**Investigation:** Liu Cheng.

**Methodology:** Feng Su, Liu Cheng.

**Resources:** Qiao Tong.

**Writing – original draft:** Feng Su, Qiao Tong.

**Writing – review & editing:** Qiao Tong.

Qiao Tong orcid: 0000-0002-9109-5265.

Feng Su orcid: 0000-0002-4989-3650.

Liu Cheng orcid: 0000-0001-6432-1717.
